# Relation of exaggerated cytokine responses of CF airway epithelial cells to PAO1 adherence

**DOI:** 10.1186/1465-9921-6-69

**Published:** 2005-07-11

**Authors:** Dianne M Kube, David Fletcher, Pamela B Davis

**Affiliations:** 1 Department of Pediatrics, Case Western Reserve University School of Medicine, BRB 8^th ^floor, 2109 Adelbert Rd. Cleveland, OH 44106, USA

**Keywords:** Cystic Fibrosis, Inflammation, *Pseudomonas aeruginosa*, IL-8, Neuraminidase, Tight junctions

## Abstract

In many model systems, cystic fibrosis (CF) phenotype airway epithelial cells in culture respond to *P. aeruginosa *with greater interleukin (IL)-8 and IL-6 secretion than matched controls. In order to test whether this excess inflammatory response results from the reported increased adherence of *P. aeruginosa *to the CF cells, we compared the inflammatory response of matched pairs of CF and non CF airway epithelial cell lines to the binding of GFP-PAO1, a strain of pseudomonas labeled with green fluorescent protein. There was no clear relation between GFP-PAO1 binding and cytokine production in response to PAO1. Treatment with exogenous aGM1 resulted in greater GFP-PAO1 binding to the normal phenotype compared to CF phenotype cells, but cytokine production remained greater from the CF cell lines. When cells were treated with neuraminidase, PAO1 adherence was equalized between CF and nonCF phenotype cell lines, but IL-8 production in response to inflammatory stimuli was still greater in CF phenotype cells. The polarized cell lines 16HBEo-Sense (normal phenotype) and Antisense (CF phenotype) cells were used to test the effect of disrupting tight junctions, which allows access of PAO1 to basolateral binding sites in both cell lines. IL-8 production increased from CF, but not normal, cells. These data indicate that increased bacterial binding to CF phenotype cells cannot by itself account for excess cytokine production in CF airway epithelial cells, encourage investigation of alternative hypotheses, and signal caution for therapeutic strategies proposed for CF that include disruption of tight junctions in the face of pseudomonas infection.

## Background

Chronic infection of the lung with *Pseudomonas aeruginosa *and the inflammatory response it stimulates cause much of the morbidity and nearly all the mortality in CF patients. Since the inflammatory response can be reduced pharmacologically in CF patients without allowing infection to increase and with benefit to the patient [1], and since infants and young children with CF have interleukin-8 (IL-8) and neutrophil count in BAL fluid significantly in excess of that observed for non-CF children with comparable bacterial burden [2,3], many investigators have concluded that the inflammatory response is excessive and deleterious in the CF lung [reviewed in [4]]. Though the cellular origin of the excessive inflammatory response in CF is not fully established, in vivo mouse CFTR complementation data suggest that the airway epithelium plays a substantive role in driving excess inflammation [5]. In many, but not all, model systems, CF airway epithelial cells respond to *P. aeruginosa *or its products with increased IL-8 and/or IL-6 production compared to non-CF cells [4,6-11]. In addition, in some, but not all, model systems binding of *P. aeruginosa *to CF airway epithelial cells is in excess of its binding to non-CF cells [12-16]. Taken together, these data have been interpreted to mean that the excess cytokine responses in CF epithelium are due to increased stimulus applied at the cell surface by elevated bacterial adherence in the CF phenotype cells [15].

Our prior studies, in two separate cell model systems, have shown that there is increase in available asialoGM1 (aGM1), which binds to *P. aeruginosa *pilin and flagellin and serves as a major ligand for this organism, on the CF member of the cell pair [17-19]. In these same cell pairs, there is an increased response of IL-8, IL-6, and granulocyte macrophage colony stimulating factor (GM-CSF) to a laboratory strain of *P. aeruginosa*, PAO1, in the CF member of the pair [6]. However, these studies did not directly address the relationship between PAO1 binding and the cytokine response. In order to test the hypothesis that the cytokine response of CF phenotype airway epithelial cells to PAO1 can be attributed solely to increased pseudomonas adherence, we took several approaches. First, we determined whether cytokine responses and *P. aeruginosa *adherence changed in parallel with increasing amounts of added PAO1. Second, we manipulated the cells to alter surface receptor access to *P. aeruginosa*. We incubated CF and non-CF cells with exogenous aGM1 to increase the binding sites for *P. aeruginosa*, and treated them with neuraminidase to add or expose more desialylated binding sites. We then compared cytokine production and binding of PAO1 in the altered cell preparations. In the cell lines that form tight junctions, we increased access to native *P. aeruginosa *binding sites on the basolateral surface by disrupting tight junctions [20], then tested the ability of the treated cells to respond to PAO1. Our results indicate that excess cytokine responses in CF airway epithelial cells do not correlate well with adherence of *P. aeruginosa*, and suggest that the excess cytokine response cannot result solely from the increased adherence of *P. aeruginosa*.

## Methods

### Cell lines

#### pCEP and pCEP-R cell lines

The development and maintenance of this matched pair of human tracheal epithelial cells derived from SV40 transformed human tracheal epithelial cells (9HTEo-, kindly provided by Dieter Gruenert, University of Calif, San Francisco) have been described previously and these methods were followed here [6,18,21].

#### 16HBE-14o- AS and S cell lines

The development and maintenance of these cell lines have been described previously and these were the methods used [6,22].

### Bacteria

The laboratory isolate PAO1 and its GFP derivative strain were kindly provided by Alice Prince, Columbia University, NY, and were grown as previously described [6].

### PAO1 Binding Assay

Green fluorescent protein (GFP)-PAO1 at 10^9 ^CFU were incubated with cell monolayers of pCEP or pCEP-R cells for 1 hr. Cells were washed with Hanks buffered salt solution (HBSS), lysed, and GFP fluorescence quantitated by fluorimeter. Serial dilutions of GFP-PAO1 were used to assess the change in GFP-PAO1 binding over a range of concentrations.

### Stimulation of cytokine production by *P. aeruginosa*

These studies were performed as previously described [6]. Briefly, 9/HTEo^- ^cells, pCEP and pCEP-R, were plated at a density of 1 × 10^6 ^cells per well on vitrogen-coated 24-well plates, and the sense and antisense clones of 16HBEo- cells were plated at density of 1 × 10^6 ^cells per 12 mm Millicell HA filter. Eighteen to 24 hr before the experiment, cells were switched to serum-free media, because PAO1 is serum-sensitive. Washed bacterial aliquots (0.5 ml/well) were incubated for 60 min with the confluent monolayers of epithelial cells at 37°C. Non-treated control wells were processed similarly with HBSS alone. For polarized 16HBE-14o- cells on filters, PAO1 and other treatments were applied to the apical surface only. As a positive control, cells were stimulated for 1 hr with IL-1β (100 ng/ml) and tumor necrosis factor (TNF)-α (100 ng/ml), (Sigma, St. Louis, MO). Cell monolayers were washed 3 times in Hanks Buffered Salt Solution (HBSS), then incubated for 24 hr in 0.5 ml serum-free cell culture medium containing 100 μg/ml gentamicin. Media were collected and analyzed for IL-8 and IL-6 by enzyme linked immunoadsorbant assay (ELISA), and normalized to the protein concentration of the lysed cells.

### Glycophospholipid addition and fluorescence microscopy

250 μg (or 5 μl of 10 mg/ml stock in dimethylsulfoxide (DMSO)) monosialoganglioside (GM1) or gangliotetraosyl ceramide (aGM1) (Matreya, Inc), was added in 0.195 ml of serum-free media for 1 hr with gentle rocking to pCEP and pCEP-RF cells. Cells were then washed twice with HBSS, and PAO1 was applied as above. Immunofluorescence was performed by incubating the cells with a 1:1000 dilution of rabbit polyclonal anti-aGM1 (Wako Pure Chemical Industries Ltd, Osaka, Japan) in phosphate buffered saline (PBS) with 0.1% bovine serum albumin (BSA), for 1 hr at 37°C, followed by two washes with PBS, and fixation with 4% paraformaldehyde (PFA) for 1 hr, and washed with PBS. Monolayers were then incubated with FITC- conjugated goat anti-rabbit antibody (Jackson Immunoresearch Laboratories Inc.) diluted 1:100 PBS with 0.1% BSA for 1 hr at room temperature, washed with PBS and fixed again with 4% PFA for 20 minutes. Cells were mounted under coverslip with Fluoromount G antifade (Southern Biotechnology Associates, Inc, Birmingham, AL) and visualized by fluorescence microsopy using a fluorescein filter set.

FITC-Peanut Agglutinin (PNA, which binds to aGM1), or FITC-Maakia Amurensis lectin (MAL I, which recognizes sialic acid in α2,3 linkages to GlcNAC), at 100 μg in 300 ml PBS, was incubated with cells for 30 minutes after fixation in 4% PFA, washed with PBS and fixed in methanol for 10 minutes. Cells were mounted under coverslip and visualized by epifluorescent microscopy with a Zeiss 100 Axiovert, 40X water immersion objective, NA 0.75, and FITC filter set. Fluorescent-conjugated lectins were purchased from Vector Laboratories, Burlingame, CA.

### Neuraminidase treatment

Neuraminidase from *Clostridium perfringens*, which removes sialic acid in α2,3, α2,6 or α2,8- linkages (5 U) or *Salmonella typhimurium *neuraminidase, which preferentially removes α2,3- linked sialic acid residues, (22.5 U) (Sigma, St. Louis, MO), was added to 200 μl serum-free media per well for 1 hr prior to PAO1 exposure. Effectiveness of treament was assessed qualitatively by immunofluorescent microscopy of fixed cells as described above.

### Treatments to disrupt tight junctions

The integrity of junctional complexes was diminished in two ways: first, by calcium chelation by incubating 16HBEo- monolayers with 30 mM EGTA in PBS buffer, for 60 min, or second, by overnight incubation with 250 μg of a monoclonal mouse E-cadherin antibody (Zymed Laboratories, San Francisco, CA) in 0.5 ml serum-free media.

### Transepithelial Resistance

Transepithelial resistance (TER) of cell monolayers grown on transwell filters was measured with a Millicell-ERS resistance system (Millipore, Bedford, MA) meter and STX-2 Electrodes (World Precision Instruments, Inc). Electrodes were equilibrated in cell culture media at room temperature, and measurements made with one electrode placed inside the insert and the other outside in the basolateral media. Baseline resistance of filters alone was determined. The TER of the polarized monolayers on filters was determined prior to treatments, immediately following treatment, and then at the final 24 hr time point.

### Cytotoxicity Assays

To quantify cytotoxity of treatments, the concentration of lactate dehydrogenase (LDH) released from cells into the medium was measured using materials purchased from Sigma Chemical Co. (St. Louis, MO) at the same time point as was used for measuring cytokines.

### Statistics

**Figure 4:**
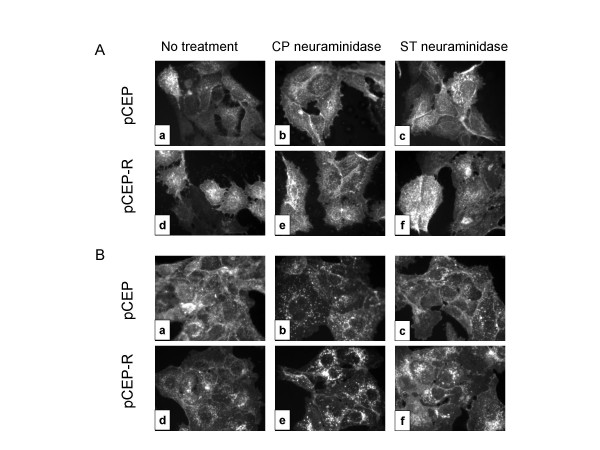
***Lectin binding to 9HTEo- cell pairs following treatment with neuraminidase***. A. FITC-PNA binding to pCEP (a-c) and pCEP-RF (d-f) cells before (a,d) and after treatment with *Clostridium perfringens *neuraminidase (b,e) or *Salmonella typhimurium *neuraminidase (c,f). Binding is similar to pCEP and pCEP-R cells after treatment. Micrographs are representative of two separate experiments. B. FITC-MALI binding to pCEP (a-c) and pCEP-RF (d-f) cells before (a,d) and after treatment with *C. perfringens *(b,e), or *S. typhimurium *neuraminidase (c,f).

**Figure 6:**
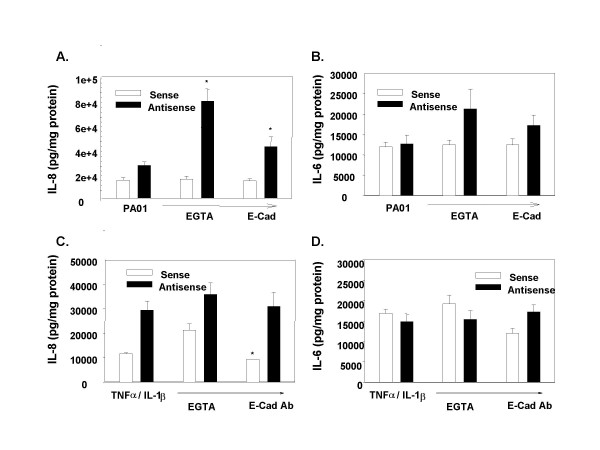
***Treatments that disrupt tight junctions increase the PAO1-stimulated IL-8 response, but not the TNF-α/IL-1β stimulated response of CF-phenotype cells***. 16HBEo- Sense (open bars) and Antisense (black bars) monolayers on filters were pretreated for 60 minutes with 30 mM EGTA prior to 1 hr. stimulation with 10^9 ^CFU PAO1/(EGTA, n = 5 independent experiments, each with triplicate wells), or an overnight incubation with 250 μg monoclonal antibody to E-Cadherin (ECAD, n = 3 independent experiments, each with triplicate wells), and the IL-8 (A, C) and IL-6 (B, D) response measured 24 H later by ELISA. The IL-8 response to PAO1 was significantly (*) increased in the 16HBE-Antisense cells following pretreatment with E-Cadherin antibody (p = 0.034) or EGTA (p < 0.001). The 16HBEo-AS cells produced significantly more IL-8 than their sense congeners (p < 0.05). There was a significant (*) reduction in the IL-6 (p = 0.05) and IL-8 (p = 0.041) response to TNF-α/IL-1β after overnight incubation to the E-cadherin antibody (n = one experiment of triplicate wells). There is a significant increase of IL-8 in response to PAO1 prior to treatment in the CF phenotype cells compared to normal (p = 0.001).

Results are expressed as mean ± standard error of the mean (SEM). All experiments reported were repeated on at least three separate occasions, and each individual cytokine experiment was performed in triplicate wells, except as specified in the legends of Figures 4 and 6. To combine multiple experiments of the 9/HTEo- cell lines, the secreted cytokine concentration (pg/mg protein) of 10^9 ^CFU of PAO1-stimulated pCEP-R cells at 24 hr. was set to 100% for each experiment, and other concentrations are expressed relative to this value. Most analysis was performed by t-test, some by ANOVA, using Sigma Plot software (SPSS, Inc., Chicago, IL). Results were considered significant when p ≤ 0.05.

## Results

### Binding of GFP-PAO1 to the cell lines

**Figure 1:**
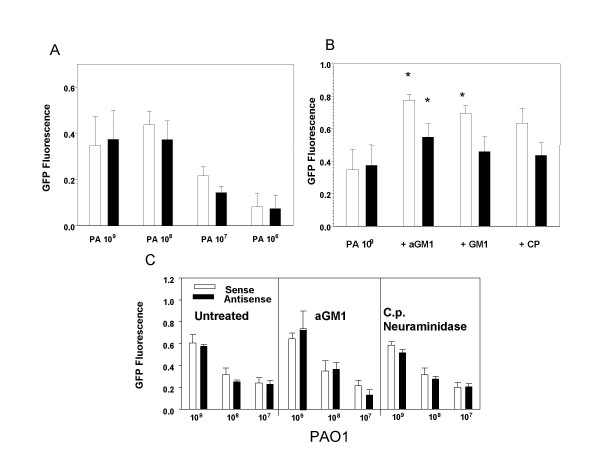
***Binding of GFP-PAO1 to airway epithelial cells***. GFP-PAO1 was added to cultured cells for one hour at 37°C, washed, and the cultures lysed and fluorescence determined (and expressed in arbitrary units). A and B, 9HTEo- cells, C, 16 HBEo- cells. For the 9HTEo- cells, binding appears to saturate at about 10^8 ^organisms/well (A) but for 16 HBEo- cells, binding increases with increasing dose of bacteria over the range tested (C). The 9HTEo- cells change GFP-PAO1 binding with addition of a GM1 or GM1, or with neuraminidase treatment (B) (*, significantly different from no treatment, p < 0.05), but the 16 HBEo- cells do not (C).

Our prior data indicate that for both the 16HBEo- AS and S cell pairs, and for the 9HTEo- pCEP and pCEP-R cell pairs, IL-8 and IL-6 production increased with addition of increasing amounts of PAO1 over the range of 10^7 ^to 10^9 ^organisms [6]. Figure 1 illustrates the changes in GFP-PAO1 binding with increasing concentrations of bacteria. For the 16 HBEo- cells, PAO1-GFP binding also increased with added PAO1 from 10^7 ^to 10^9 ^CFU/mL, but for 9HTEo- cells, binding increased from 10^6 ^to 10^8 ^CFU/mL but did not increase not further with 10^9 ^CFU/mL, even though the cytokine responses did. Binding of GFP-PAO1 was similar in untreated 16HBEo- sense (S) and antisense (AS) cell lines, at all concentrations, and in untreated 9HTEo-pCEP and pCEP-R cell lines, at all concentrations (Figure 1). Therefore, the previously reported increase in available aGM1 in the CF member of the pairs, confirmed below, was not necessarily associated with increased GFP-PAO1 binding, and increased cytokine production was not invariably associated with increased binding of GFP-PAO1.

### Providing additional *P. aeruginosa *binding sites by addition of asialoGM1

**Figure 2:**
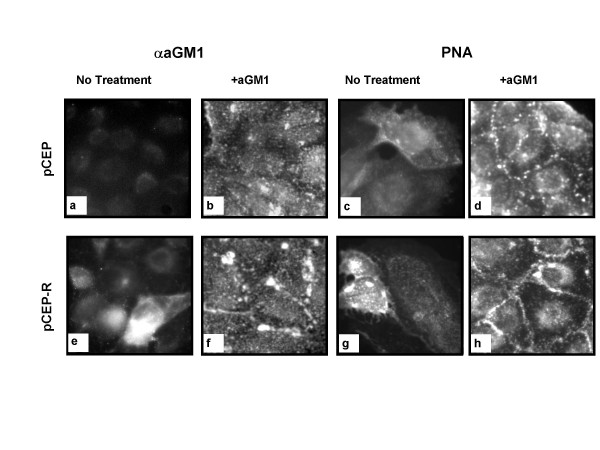
***Exogenous aGM1 is incorporated into 9HTEo-pCEP and pCEP-R***. pCEP (a-d) and pCEP-R (e-h) cells were incubated with either vehicle (a,c,e,g) or aGM1 (b,d,f,h) and fluorescent staining with either antibody to aGM1 and FITC secondary (b,f) or FITC- conjugated PNA (D,H) was performed. In the untreated state, there is more binding of antibody to aGM1 or PNA to pCEP-R than pCEP cells. After incubation with aGM1, fluorescence patterns are similar for pCEP and pCEP-R cells with antibody to aGM1 (b,f) and PNA (d,h). Micrographs are representative of 3 separate experiments.

**Figure 3:**
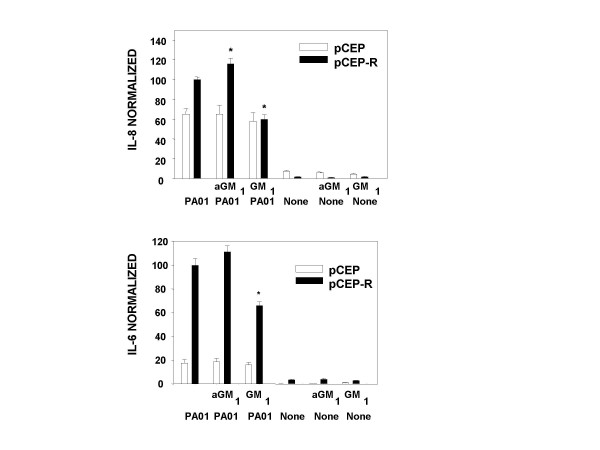
***IL-8 and IL-6 responses to PAO1 or no stimulation, with or without preincubation with aGM1 or GM1***. *, different from no treatment, p < 0.05.

Others report that exogenous aGM1 is incorporated into the cell membrane and provides additional binding sites for *P. aeruginosa *[23]. We therefore incubated our cell lines with exogenous a GM1 and measured cell-associated aGM1, GFP-PAO1 binding, and cytokine responses. Incubation of the 9/HTEo- cell lines with aGM1 resulted in increased cell-associated aGM1, as demonstrated both by specific antibody binding, and by binding of PNA, a lectin which recognizes aGM1 (Figure 2). There was no change in LDH release (Table 1). Prior to treatment, as reported previously [19], the 9HTEo-pCEP-R cells displayed more aGM1 than the 9HTEo-pCEP cells (Figure 2A vs E for aGM1 and C vs G for PNA), but following treatment, the two cell lines had similar aGM1 antibody fluorescence and PNA fluorescence (Figure 2, B vs F and D vs H). Prior to treatment, binding of GFP-PAO1 to the two cell types is equivalent, (Figure 1, Table 1). After aGM1 incubation, both cell lines showed increased GFP-PAO1 binding, but more so in the non-CF than the CF phenotype cells (Table 1). Untreated CF phenotype cells had increased IL-8 and IL-6 production in response to PAO1 compared to normal, as previously reported [6]. Following incubation, although aGM1 and PAO1 binding increased in the normal cells, cytokine production did not, but IL-8 production by the CF phenotype cells showed a statistically significant increase (Figure 3). As a control, the cells were loaded with GM1, which is less efficient in binding PAO1. Following GM1 preincubation, despite the increase in PAO1-GFP binding (Table 1), there was a significant decrease in production of both IL-8 and IL-6 by the CF phenotype cell line (Figure 3), possibly because more PAO1 was bound at sites that do not initiate an inflammatory signal. No changes in cytokine response to TNF-α/IL-1β occurred following incubation with aGM1 or GM1 (data not shown).

**Table 1:** Binding of PAO1 and LDH release by 9HTEo- cell lines

	**pCEP**		**pCEP-R**	
	
	LDH (U/ml)	GFP -PA01 (RFU)	LDH (U/ml)	GFP-PA01 (RFU)
PA	8.83 ± 2.5	0.3483 ± 0.12	4.64 ± 0.7*	0.3745 ± 0.123
PA + EGTA	3.77 ± 0.9	ND	2.82 ± 0.0	ND
PA + aGM1	2.36 ± 0.5	0.7764 ± 0.037†	3.3 ± 0.5	0.5489 ± 0.085*†
PA + GM1	2.83 ± 0.0	0.6929 ± 0.049†	1.89 ± 0.0	0.4598 ± 0.090*
PA + C.p.	ND	0.6350 ± 0.091†	ND	0.4359 ± 0.079*
No treatment	4.79 ± 1.0	ND	2.44 ± 0.9 *	ND
EGTA alone	2.36 ± 0.5	ND	4.24 ± 0.5	ND
aGM1 alone	3.3 ± 0.5	ND	2.83 ± 0.9	ND
GM1 alone	2.83 ± 0.9	ND	3.3 ± 0.5	ND

*Different from pCEP congener, p < 0.05

† Different from no treatment, p < 0.05

In polarized epithelial cell lines (16BHBEo-), the addition of aGM1 or GM1 did not increase GFP-PAO1 binding (Table 2, Figure 1), nor alter the proinflammatory cytokine response to *P. aeruginosa *or TNF-α/IL-1β (data not shown).

**Table 2:** Transepithelial resistance, LDH release and PAO1 binding to 16HBEo- cell lines.

	**Sense**			**AntiSense**		
	
	Resistance (Ω*cm^2^)	LDH (u)	GFP-PA01 (RFU)	Resistance (Ω*cm^2^)	LDH (u)	GFP-PA01 (RFU)
PA alone	229.0 ± 13.8	4.08 ± 0.4	0.6056 ± 0.078	217.85 ± 8.0	2.12 ± 0.2*	0.5753 ± 0.021
PA + EGTA	136.14 ± 6.1	2.42 ± 0.3	ND	142.43 ± 5.9	2.78 ± 0.6	ND
PA + ECAD	126.0 ± 2.3	2.95 ± 0.3	ND	131.0 ± 1.3	2.99 ± 0.4	ND
PA + aGM1	ND	ND	0.6456 ± 0.051	ND	ND	0.7437 ± 0.165†
PA + C.p.	ND	ND	0.5851 ± 0.031	ND	ND	0.5154 ± 0.031
No treatment	316.54 ± 10.5	4.31 ± 0.7	ND	225.72 ± 2.9	1.24 ± 0.1*	ND
EGTA alone	213.58 ± 15.2	3.22 ± 0.0	ND	202.4 ± 4.3	0.86 ± 0.0	ND
ECAD alone	147.186 ± 14.4	3.73 ± 0.0	ND	151.09 ± 6.2	1.07 ± 0.2	ND

* Different from Sense congener, p < 0.05

†Different from no aGM1, p < 0.05

### Providing additional *P. aeruginosa *binding sites by enzymatic removal of sialic acid

**Figure 5:**
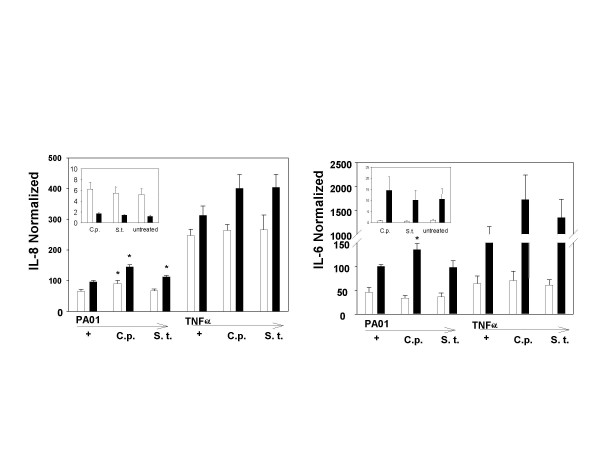
***Neuraminidase treatment alters cytokine responses in 9HTEo- cell lines***. IL-8 (A) and IL-6 (B) responses to 10^9 ^CFU PAO1 or TNF-α/IL-1β are shown. For 9HTEo-pCEP cells, only IL-8 secretion increased, and only following treatment with *C. perfringens *neuraminidase (C.p.), not with the enzyme from *S. typhimurium*. However, 9HTEo-pCEP-R cells showed increased IL-8 response to PAO1 following treatment with either enzyme and IL-6 response to C.p. neuraminidase. Three separate experiments were performed, each with triplicate wells. (*, different from untreated samples, p < 0.05).

*C. perfringens *neuraminidase removes sialic acid in the α2,3, α2,6, and α2,8 linkages. *S. typhimurium *neuraminidase attacks the α2,3 linkage preferentially. Increases in binding of PNA, which recognizes aGM1, are evident for both cell lines following neuraminidase treatment (Figure 4A, panels B,C,E,F). This increase may result from relief of steric hindrance to binding to existing sites, since some investigators find that the final sialic acid residue is not removed by their action. Nevertheless, MAL I, a lectin which recognizes sialic acid in terminal α2,3 linkages, shows visible decrease in surface binding in the non-CF phenotype cells that have been treated with *C. perfringens *neuraminidase (Figure 4B, panels B and E), but the change in MAL I fluorescence after *S. typhimurium *neuraminidase is less clear (Figure 4B, panels E and F). *Clostridium perfringens *neuraminidase treatment significantly increased GFP-PAO1 binding on the non-CF cell line (Table 1), but the cytokine responses to PAO1 or TNF-α/IL-1β did not increase in the non-CF cells (Figure 4). There was no significant increase in GFP-PAO1 binding in the CF phenotype cells, even though they showed increased IL-8 and IL-6 responses following treatment with the broad spectrum neuraminidase of *C. perfringens*. Following treatment with the more specific *S. typhimurium *enzyme only IL-8 was increased (Figure 5).

*C. perfringens *neuraminidase treatment did not alter either the IL-8 response or PAO1 binding in the polarized cell lines. However, the IL-6 response of the CF phenotype line was reduced (data not shown).

### Exposure of basolateral receptors to *P. aeruginosa*

We expected that disrupting the tight junctions in the monolayer would permit PAO1, applied to the apical surface, to access basolateral receptors that were not available when the monolayer was intact [23], and thereby would increase the cytokine response to PAO1. The tight junctions in both the Sense (control) and Antisense-treated (CF phenotype) 16 HBEo- cell lines were disrupted by treatment with EGTA or antibodies to E-cadherin, as shown by the decrease in transepithelial resistance following these treatments (Table 2). Incubation of the filters without disrupting agents for the time course of the experiment did not alter transepithelial resistance. When incubation with the disrupting agents was combined with *P. aeruginosa *exposure, the transepithelial resistance fell even further, to approximate that of the filters alone. The non CF phenotype cell lines (pCEP and 16HBEo- Sense) show both a greater transepithelial resistance and a greater amount of lactate dehydrogenase in the medium at baseline than CF phenotype cell lines (pCEP-R and 16HBEo- AntiSense) (Tables 1 and 2). Apoptosis is reported to be reduced in CF versus non-CF cell lines [24,25], which may account for the lesser release of LDH. However, none of the treatments that alter PA receptor availability further disrupted the integrity of cellular membranes or increased LDH release (Table 1).

Although the disruptive treatments had similar effects on resistance in CF and nonCF phenotype cells, the cytokine response to PAO1 increased with disruption of tight junctions only in the CF phenotype cells. There was no increase in cytokine production following TNF-α/IL-1β stimulation: in fact in one sample a small decrease was seen (Figure 6). In order to test whether the EGTA treatment, in and of itself, altered cytokine production by airway epithelial cell lines, we treated non-polarized 9HTEo- cell lines with EGTA in the same manner as it was applied to the 16HBEo- cells. There was a slight but statistically significant decrease in IL-6 in response to PAO1 production by the CF phenotype cells, but no other changes (data not shown). Since in the polarized cells, EGTA pretreatment resulted in increased cytokine production in the CF cell line, and if anything, EGTA treatment of nonpolarized cells produced no such increase, we ascribe the increases in the polarized cells to disruption of the tight junctions and not to some nonspecific effect of EGTA.

## Discussion

**Figure 7:**
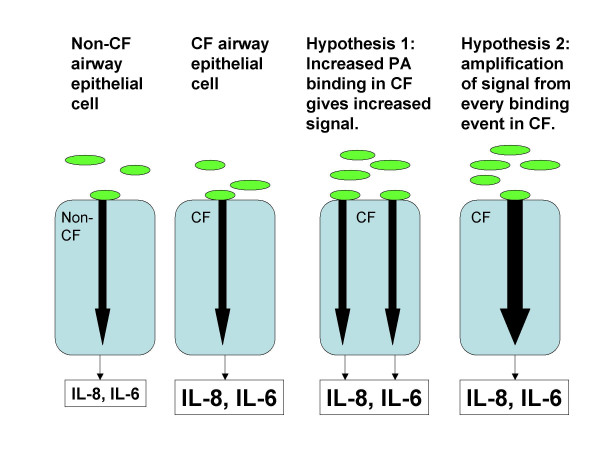
***Cartoon comparing CF and non-CF epithelial cell responses to P. aeruginosa and illustrating two hypotheses to explain the increased cytokine response from CF airway epithelial cells***. Bacterial adherence to the cell stimulates an intracellular signaling cascade. CF cells produce more IL-8 and IL-6 than non-CF cells. In the first hypothesis, increased bacterial adherence to the CF cell leads to increased signal, with consequent increase in IL-8 and IL-6 secretion. In the second hypothesis, the CF cell responds to each binding event with amplification of the signal compared to non-CF cells, and increased IL-8 and IL-6 secretion.

In some model systems, CF airway epithelial cells produce more IL-8 and/or IL-6 than non CF cells in response to *P. aeruginosa*, and in some model systems, CF cell surfaces bind the organism to a greater extent than normal [6-16]. The studies reported here were designed to test the hypothesis that increased binding sites for PAO1 result in increased stimulus and increased cytokine production in response to PAO1 in airway epithelial cells (Figure 7). The hypothesis was not supported. Surprisingly, although aGM1 was increased on the CF phenotype cells studied here under basal conditions, GFP-PAO1 binding was not, so the increased cytokine responses of CF phenotype cells to PAO1 in the basal state [6] cannot be attributed solely to increased PAO1 adherence. Moreover, increasing the binding of PAO1 to non-polarized normal airway epithelial cell lines (9HTEo-pCEP), either by adding aGM1 or by cleaving sialic acid at the cell surface, does not change the cytokine responses to PAO1. CF phenotype cells (9HTEo-pCEP-R) still respond to PAO1 with greater cytokine release than their matched normal counterparts, despite significantly less PAO1 adherence than normal phenotype cells. For matched polarized cell lines (16HBEo-), there was little change in PAO1 binding from adding aGM1 or cleaving sialic acid at the cell surface in either the CF or the non CF line, and little change in the cytokine response to PAO1. However, when the basolateral surface was made available for PAO1 binding by disruption of the tight junctions, cytokine responses to PAO1 increased only in the CF phenotype cells.

It is likely that there are multiple ligands for PAO1 on airway epithelial cells. Two that have been identified are aGM1 and CFTR itself [18,24], and it is likely that GM1 is a weak binding site as well. Thus, it is possible that GFP-PAO1 adheres more to increased aGM1 binding sites on the CF cells (which apparently signal for inflammatory mediators) but may adhere less at other sites, perhaps at CFTR itself, making it appear that adherence has little relation to cytokine response when in fact a only a subset of pseudomonas receptors is responsible for the increased response. Nevertheless, attempts to increase aGM1 directly did not produce the expected changes in the cytokine responses of non-CF cell lines, but did enhance the responses of the CF cell lines. Adding exogenous aGM1 effectively equalized surface aGM1 in both normal and CF cell lines as measured by antibodies to aGM1 or PNA lectin binding, and actually increased GFP-PAO1 binding to the non CF relative to the CF cell line. Were *P. aeruginosa *binding to aGM1 the principal determinant of the pro-inflammatory cytokine response, one would expect that the response of the normal cell lines under these conditions would equal or exceed that of the CF phenotype cell lines. However, the CF cell lines still produced more IL-8 in response to PAO1. Although one could argue that increasing aGM1 in this manner might produce binding sites that are not connected to the signaling machinery, others have shown that exogenous aGM1 incorporates into cellular membranes, increases the binding of *P. aeruginosa*, and augments biological responses to *P. aeruginosa*, including cytotoxicity, internalization and the apoptotic response [23]. Moreover, the CF cell line treated in this manner did augment its cytokine response to PAO1 (but not to another stimulus, TNF-α/IL-1β, eliminating the possibility of a generalized increase in cytokine production). In contrast, GM1 preincubation, which also resulted in increased PAO1 binding, did not increase the IL-8 response to PAO1: in this case, the increase in binding of *P. aeruginosa *in the GM1-incubated cells is probably not coupled to a proinflammatory signaling cascade. Association of PAO1 with a non-signaling GM1 ligand could block access to aGM1 receptors, actually reducing the response.

Another attempt to alter access to aGM1 binding sites also did not reveal association between binding and inflammatory response. Treating the cells with the broad spectrum neuraminidase from *C. perfringens *resulted in significantly increased lectin binding sites and IL-8 response to PAO1 (but not to TNF-α/IL-1β) in both CF phenotype and non-CF phenotype 9HTEo- cells. However, the excess cytokine response of the CF cells was preserved following neuraminidase treatment, despite equalizing apparent binding of PAO1. Neuraminidase from *S. typhimurium*, which preferentially removes sialic acid in the α2,3 linkage, produced a significant increase in IL-8 production in response to PAO1 only in the CF phenotype cells.

We made no attempt to assess with lectin binding or specific antibody the nature of the basolateral binding sites revealed in polarized cultures by disruption of tight junctions. Others have shown that allowing access to basolateral receptors greatly increases *P. aeruginosa *binding, cytotoxicity, internalization, and apoptosis independent of CFTR [23,26,27]. Nevertheless, opening tight junctions did not enhance PAO1-stimulated cytokine production in the non-CF cell line, whereas it did in the CF congener.

## Conclusion

The data presented here indicate that the increased cytokine responses in CF airway epithelial cells to *P. aeruginosa *cannot be attributed solely to increased adherence of the organism. There are several implications of this finding. First, these data focus attention on alternative hypotheses to explain the increased inflammatory response of the CF airway epithelial cell (Figure 7). Our data make the hypothesis that increased pseudomonas binding entirely accounts for the increased inflammatory response of CF epithelium [15] much less likely. Alternatively, there may be increased amplification of the signal from the bacterium in CF cells to account for the increased response. Considerable attention has been paid to the excess activation of NF-κB in CF epithelial cells. Some investigators find that there is activation of this transcription factor even in the unstimulated state in CF epithelial cells, and others find that it is activated to excess only under conditions of stimulation. This pivotal transcription factor could account for a panoply of abnormalities, including the excess cytokine production documented here, but also increased release of MMP-9 and reduced apoptosis of CF airway epithelial cells. Others have proposed that in CF there is failure of anti-inflammatory control mechanisms such as IL-10, NO, or transcription factors that compete with NF-κB for helicases, or there may be subtle abnormalities in both the pro- and anti-inflammatory arms of the cascade [reviewed in [4]]. A second caveat raised by our data is that disrupting tight junctions in the CF epithelium can markedly increase inflammatory responses to *P. aeruginosa*, even if the bacteria are applied only briefly and the cells are given opportunity to recover. Moreover, the combination of disrupting agents and *P. aeruginosa *produced complete loss of the electrophysiologic barrier in a manner that EDTA or anti-E-cadherin did not. These observations signal caution for therapeutic strategies that propose to access the basolateral surface of CF airway epithelial cells by disrupting the tight junctions in vivo [28,29], especially in patients already infected with *P. aeruginosa*.

## Competing interests

The author(s) declare that they have no competing interests.

## Authors' contributions

DMK designed the experiments, conducted many of them, prepared the figures, and drafted the paper. DF conducted some of the experiments, including stimulations of cells for cytokine measurements and determinations of transepithelial resistance. PBD contributed to experimental design and data interpretation and wrote and edited the final version of the paper. All authors read and approved the final manuscript.

## References

[B1] Konstan MW, Byard PJ, Hoppel CL, Davis PB (1995). Effect of high-dose ibuprofen in patients with cystic fibrosis. N Engl J Med.

[B2] Muhlebach MS, Reed W, Noah TL (2004). Quantitative cytokine gene expression in CF airway. Pediatr Pulmonol.

[B3] Muhlebach MS, Stewart PW, Leigh MW, Noah TL (1999). Quantitation of inflammatory responses to bacteria in young cystic fibrosis and control patients. Am J Respir Crit Care Med.

[B4] Chmiel JF, Davis PB (2003). State of the Art: Why do the lungs of patients with cystic fibrosis become infected and why can't they clear the infection?. Respir Res.

[B5] Oceandy D, McMorran BJ, Smith SN, Schreiber R, Kunzelmann K, Alton EW, Hume DA, Wainwright BJ (2002). Gene complementation of airway epithelium in the cystic fibrosis mouse is necessary and sufficient to correct the pathogen clearance and inflammatory abnormalities. Hum Mol Genet.

[B6] Kube D, Sontich U, Fletcher D, Davis PB (2001). Proinflammatory cytokine responses to P. aeruginosa infection in human airway epithelial cell lines. Am J Physiol Lung Cell Mol Physiol.

[B7] DiMango E, Ratner AJ, Bryan R, Tabibi S, Prince A (1998). Activation of NF-kappaB by adherent *Pseudomonas aeruginosa *in normal and cystic fibrosis respiratory epithelial cells. J Clin Invest.

[B8] Joseph T, Look D, Ferkol T (2005). NF-kappaB activation and sustained IL-8 gene expression in primary cultures of cystic fibrosis airway epithelial cells stimulated with Pseudomonas aeruginosa. Am J Physiol Lung Cell Mol Physiol.

[B9] Becker MN, Sauer MS, Muhlebach MS, Hirsh AJ, Wu Q, Verghese MW, Randell SH (2004). Cytokine secretion by cystic fibrosis airway epithelial cells. Am J Respir Crit Care Med.

[B10] Escotte S, Tabary O, Dusser D, Majer-Teboul C, Puchelle E, Jacquot J (2003). Fluticasone reduces IL-6 and IL-8 production of cystic fibrosis bronchial epithelial cells via IKK-beta kinase pathway. Eur Respir J.

[B11] Tabary O, Zahm JM, Hinnrasky J, Couetil JP, Cornillet P, Guenounou M, Gaillard D, Puchelle E, Jacquot J (1998). Selective up-regulation of chemokine IL-8 expression in cystic fibrosis bronchial gland cells in vivo and in vitro. Am J Pathol.

[B12] Saiman L, Prince A (1993). *Pseudomonas aeruginosa *pili bind to asialoGM1 which is increased on the surface of cystic fibrosis epithelial cells. J Clin Invest.

[B13] Zar H, Saiman L, Quittell L, Prince A (1995). Binding of *Pseudomonas aeruginosa *to respiratory epithelial cells from patients with various mutations in the cystic fibrosis transmembrane regulator. J Pediatr.

[B14] Scheid PL, Kempster U, Griesenbach JC, Davies A, Dewar PP, Weber WH, Colledge MJ, Evans D, Geddes M, Alton EW (2001). Inflammation in cystic fibrosis airways: relationship to increased bacterial adherence. Eur Respir J.

[B15] Davies J, Dewar A, Bush A, Pitt T, Gruenert D, Geddes DM, Alton EW (1999). Reduction in the adherence of *Pseudomonas aeruginosa *to native cystic fibrosis epithelium with anti-asialoGM1 antibody and neuraminidase inhibition. Eur Respir J.

[B16] Davies JC, Stern M, Dewar A, Caplen NJ, Munkonge FM, Pitt T, Sorgi F, Huang L, Bush A, Geddes DM, Alton EW (1997). CFTR gene transfer reduces the binding of Pseudomonas aeruginosa to cystic fibrosis respiratory epithelium. Am J Respir Cell Mol Biol.

[B17] Bryan R, Kube D, Perez A, Davis P, Prince A (1998). Overproduction of the CFTR R domain leads to increased levels of asialoGM1 and increased *Pseudomonas aeruginosa *binding by epithelial cells. Am J Respir Cell Mol Biol.

[B18] McNamara N, Khong A, McKemy D, Caterina M, Boyer J, Julius D, Basbaum C (2001). ATP transduces signals from ASGM1, a glycolipid that functions as a bacterial receptor. Proc Natl Acad Sci USA.

[B19] Kube D, Adams L, Perez A, Davis PB (2001). Terminal sialylation is altered in airway cells with impaired CFTR-mediated chloride transport. Am J Physiol Lung Cell Mol Physiol.

[B20] Lee A, Chow D, Haus B, Tseng W, Evans D, Fleiszig S, Chandy G, Machen T (1999). Airway epithelial tight junctions and binding and cytotoxicity of *Pseudomonas aeruginosa*. A J Physiol.

[B21] Perez A, Risma KA, Eckman EA, Davis PB (1996). Overexpression of R domain eliminates cAMP-stimulated Cl- secretion in 9/HTEo- cells in culture. Am J Physiol.

[B22] Rajan S, Cacalano G, Bryan R, Ratner AJ, Sontich CU, van Heerckeren A, Davis PB, Prince A (2000). *Pseudomonas aeruginosa *induction of apoptosis in respiratory epithelial cells: analysis of the effects of cystic fibrosis transmembrane conductance regulator dysfunction and bacterial virulence factors. Am J Respir Cell Mol Biol.

[B23] Comolli JC, Waite LL, Mostov KE, Engel JN (1999). Pili binding to asialo-GM1 on epithelial cells can mediate cytotoxicity or bacterial internalization by *Pseudomonas aeruginosa *. Infect Immun.

[B24] Cannon CL, Kowalski MP, Stopak KS, Pier GB (2003). Pseudomonas aeruginosa-induced apoptosis is defective in respiratory epithelial cells expressing mutant cystic fibrosis transmembrane conductance regulator. Am J Respir Cell Mol Biol.

[B25] Gottlieb RA, Dosanjh A (1996). Mutant cystic fibrosis transmembrane conductance regulator inhibits acidification and apoptosis in C127 cells: possible relevance to cystic fibrosis. Proc Natl Acad Sci USA.

[B26] Plotkowski MC, De Bentzmann S, Pereira SH, Zahm JM, Bajolet-Laudinat O, Roger P, Puchelle E (1999). *Pseudomonas aeruginosa *internalization by human epithelial respiratory cells depends on cell differentiation, polarity, and junctional complex integrity. Am J Respir Cell Mol Biol.

[B27] Fleiszig SM, Evans DJ, Do N, Vallas V, Shin S, Mostov KE (1997). Epithelial cell polarity affects susceptibility to Pseudomonas aeruginosa invasion and cytotoxicity. Infect Immun.

[B28] Parsons DW, Grubb BR, Johnson LG, Boucher RC (1998). Enhanced in vivo airway gene transfer via transient modification of host barrier properties with a surface-active agent. Hum Gene Ther.

[B29] Coyne CB, Kelly MM, Boucher RC, Johnson LG (2000). Enhanced epithelial gene transfer by modulation of tight junctions with sodium caprate. Am J Respir Cell Mol Biol.

